# Association between site of infection and in-hospital mortality in patients with sepsis admitted to emergency departments of tertiary hospitals in Medellin, Colombia

**DOI:** 10.5935/0103-507X.20190011

**Published:** 2019

**Authors:** César Caraballo, Johana Ascuntar, Carolina Hincapié, Camilo Restrepo, Elisa Bernal, Fabián Jaimes

**Affiliations:** 1 Grupo Académico de Epidemiología Clínica, Universidad de Antioquia - Medellín, Colombia.; 2 Servicio de Medicina Interna, Hospital Pablo Tobón Uribe - Medellín, Colombia.; 3 Dirección de Investigaciones, Hospital San Vicente Fundación - Medellín, Colombia.; 4 Center for Outcomes Research and Evaluation, Yale University School of Medicine - New Haven, CT, USA.

**Keywords:** Sepsis, Septic shock, Shock, Mortality, Prognosis, Infection, Intensive care

## Abstract

**Objective:**

To determine the association between the primary site of infection and in-hospital mortality as the main outcome, or the need for admission to the intensive care unit as a secondary outcome, in patients with sepsis admitted to the emergency department.

**Methods:**

This was a secondary analysis of a multicenter prospective cohort. Patients included in the study were older than 18 years with a diagnosis of severe sepsis or septic shock who were admitted to the emergency departments of three tertiary care hospitals. Of the 5022 eligible participants, 2510 were included. Multiple logistic regression analysis was performed for mortality.

**Results:**

The most common site of infection was the urinary tract, present in 27.8% of the cases, followed by pneumonia (27.5%) and intra-abdominal focus (10.8%). In 5.4% of the cases, no definite site of infection was identified on admission. Logistic regression revealed a significant association between the following sites of infection and in-hospital mortality when using the urinary infection group as a reference: pneumonia (OR 3.4; 95%CI, 2.2 - 5.2; p < 0.001), skin and soft tissues (OR 2.6; 95%CI, 1.4 - 5.0; p = 0.003), bloodstream (OR 2.0; 95%CI, 1.1 - 3.6; p = 0.018), without specific focus (OR 2.0; 95%CI, 1.1 - 3.8; p = 0.028), and intra-abdominal focus (OR 1.9; 95%CI, 1.1 - 3.3; p = 0.024).

**Conclusions:**

There is a significant association between the different sites of infection and in-hospital mortality or the need for admission to an intensive care unit in patients with sepsis or septic shock. Urinary tract infection shows the lowest risk, which should be considered in prognostic models of these conditions.

## INTRODUCTION

Sepsis is a systemic response secondary to an infectious process that causes organ dysfunction that endangers life;^(^^1,2^^)^ therefore, timely diagnosis and treatment are essential. It has been estimated that 35 million people are diagnosed with this condition each year, with 6 million dying in the same period.^(^^3^^)^

In 2001, a hypothetical model was proposed for the staging of patients with sepsis, similar to the TNM model used in cancer patients,^(^^4^^)^ and was named PIRO ("P": predisposition to infections due to conditions such as drug-induced immunosuppression, AIDS, or age; "I": characteristics of the infection such as etiology, site, or presence of bacteremia; "R": characteristics of the response such as systemic inflammation, shock, or other; and "O": organ dysfunction). Although PIRO continues to be a theoretical concept rather than a tool for daily clinical practice, some studies have been conducted using its variables to predict the prognosis of patients with sepsis in different scenarios, having found, in general, that each component (P, I, R, and O) predicts mortality independently.^(^^5-8^^)^ However, when analyzing the variables of the infection component, the site of infection has not been found to be equally constant as a prognostic factor.^(^^9^^)^ Different scores or scales have been proposed to determine the severity of this disease and its prognosis for use in the emergency department,^(^^2,5,10-12^^)^ but only the Mortality in Emergency Department Sepsis (MEDS) score considers the source of the infection.^(^^11^^)^

Estimating the strength of the association between site of infection and prognosis of the sepsis patient could be an additional tool for the attending medical staff to make relevant clinical decisions by estimating individual's risk with greater accuracy. Additionally, in the context of a vaguely defined clinical problem,^(^^2,4,13^^)^ identifying prognostic differences according to the site of infection may allow a better characterization and definition of sepsis. Considering the above, the main objective of our study was to determine the association between the primary site of infection and the mortality of patients with sepsis in the emergency department. The secondary objective was to determine the association between the site of infection and the need for admission to the intensive care unit (ICU).

## METHODS

This was a secondary analysis of data obtained from a multicenter prospective cohort study (*Análisis instrumental del protocolo de reanimación con metas tempranas en pacientes con sepsis grave en el servicio de urgencias* (Instrumental analysis of the early goal-directed resuscitation protocol in patients with severe sepsis in the emergency department). COLCIENCIAS-UdeA: 111556933362; Contract No. 580-2013). The objective of the study was to determine the effect of each early goal-directed resuscitation strategy and the effect of antibiotics on in-hospital mortality. The study was approved by the ethics committee of the institution (Bioethics Committee, Institute of Medical Research, Universidad de Antioquia, Act 008/17 of May/2012).

The study was conducted in the emergency departments and ICUs of three tertiary care university hospitals in Medellin (Colombia): *Hospital Universitario San Vicente Fundación* (HUSVF, 560 adult beds and 45 ICU beds in 4 units), *Hospital Pablo Tobón Uribe* (HPTU, 360 adult beds and 40 ICU beds in 3 units), and *IPS Universitaria León XIII* (IPSU, 450 adult beds and 24 ICU beds in 2 units). The study was approved by the ethics committees of the three institutions, and informed consent was requested from all participants. The period of patient data collection was between June 1, 2014 and February 29, 2017.

Under the international definitions established at the beginning of participant recruitment, the study included patients older than 18 years who were hospitalized in the emergency department with a recorded diagnosis of severe sepsis or septic shock. Severe sepsis was defined as suspected or confirmed infection with at least two criteria of the systemic inflammatory response syndrome and one of the following criteria for organ dysfunction: Glasgow < 15; PaO_2_/FiO_2_ < 300 or need for mechanical ventilation; urinary output < 0.5mL/kg/h for 2 hours reported in the clinical history; creatinine > 2mg/dL without previous history of renal disease or increase of 0.5mg/dL with respect to previous values; international normalized ratio (INR) > 1.5 or partial thromboplastin time (PTT) > 60 seconds; ileus (described in the clinical history); platelets < 150,000 cells/mm^3^; total bilirubin > 2mg/dL; hyperlactatemia > 2mmol/L; capillary filling: slow or greater than 2 seconds; systolic blood pressure < 90mmHg or mean arterial pressure < 70mmHg during the first 6 hours after admission.

Exclusion criteria included refusal by the patient, their family, or attending physician to participate in the study; concurrent diagnoses of pregnancy, myocardial infarction, cerebrovascular event, asthmatic crisis, arrhythmia, trauma, gastrointestinal bleeding, seizures not associated with meningitis, overdose of psychoactive substances, need for surgery in the first 24 hours, burns, CD4 count < 50 cells per mm^3^, hyperosmolar state or diabetic ketoacidosis, or cirrhosis; discharge or remission in the first 24 hours of hospitalization; previous participation in the study; referral from another institution where patients had been hospitalized for over 24 hours; or a do-not-resuscitate order.

### Definition of variables

#### Site of infection

According to the suspected site of infection on admission to the emergency department, after assessment by the coinvestigator in charge, the definition of the source of infection in each patient was standardized according to Center for Disease Control (CDC) criteria.^(^^14^^)^ These sites were grouped as urinary tract, lower respiratory tract (pneumonia), intra-abdominal, bloodstream, and skin and soft tissue infection, as well as infection of other sites and infection without focus. This last group included those patients whose clinical diagnosis was sepsis but in whom the primary site of infection could not be determined despite clinical, imaging and paraclinical examination.

#### Potential confounding variables

The following criteria were considered as adequate treatment: intravenous fluids, at least 1500cc of crystalloids in the first hour, starting antibiotics in the first three hours, and taking blood cultures in the first three hours. Additionally, all the procedures and treatments of the original Rivers protocol performed during the first 24 hours of hospital stay were recorded.

Comorbidities were considered using the Charlson index.^(^^15^^)^ Sepsis severity was assessed using the Sequential Organ Failure Assessment (SOFA)^(^^16^^)^ and the Acute Physiology and Chronic Health Evaluation II (APACHE II),^(^^17^^)^ along with lactate levels on admission to the emergency department. These scores were estimated based on the data obtained in the first 6 hours after admission. Laboratory results necessary to estimate these scores that were either missing or not requested were assumed to be normal.

#### Outcomes

In-hospital mortality was the primary outcome, and the length of hospital stay and need for admission to the ICU were considered secondary outcomes.

#### Data source

Research assistant nurses trained in each institution performed the entire process of patient screening and selection, as well as data collection using a standardized form. The co-investigators continuously reviewed and monitored the included patients and the data collected by the assistants. To identify patients, all those admitted to the emergency department with a diagnosis of infection, sepsis, severe sepsis, or shock were screened. The definitions of the source of infection and the presence of organ dysfunction or shock were verified based on the data extracted from the medical records in the first 6 hours. All data related to the diagnosis and treatment (including time) were also extracted from the medical records.

#### Statistical analysis

Continuous variables were described using medians and interquartile ranges, or the mean and standard deviation, according to their distribution. Categorical variables were described as proportions. Continuous variables were compared between groups using the Kruskal-Wallis test, and categorical variables were compared with the chi-squared test.

To determine the association between outcomes and type of infection, a logistic regression model was performed, and three subsequent sequential models were used. The confounding variables of age, sex, and Charlson index were included in the first model. For the second model, the following variables were added: intravenous fluid therapy in the first hour ≥ 1500mL, starting antibiotics in the first three hours, and taking blood cultures in the first three hours. For the third model, the SOFA score, APACHE II score, and lactate levels were added. The same analysis was performed with the "need for ICU admission" outcome. Measures of association (odds ratio - OR) are accompanied by their corresponding 95% confidence intervals (95%CI). The possibility of interaction was not considered, and multicollinearity between variables was ruled out by variance inflation factor cutoff values below 10.^(^^18^^)^ All statistical analyses were performed with STATA V.14 software.

## RESULTS

**Figure 1:**
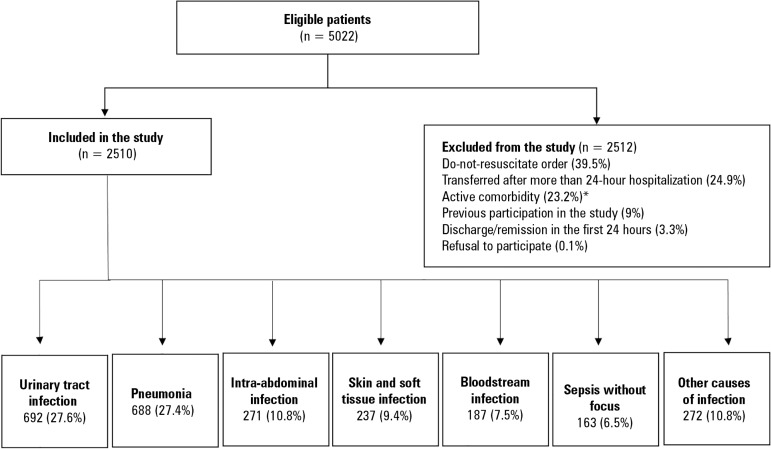
Study population. * Cirrhosis (n = 89; 23.7%), surgery < 24 hours (n = 72; 19.2%), gastrointestinal bleeding (n = 58; 15.5%), CD4 count < 50 cells/mm^3^ (n = 52; 13.9%), diabetic ketoacidosis or hyperosmolar state (n = 47; 12.5%), seizures not due to meningitis (n = 26; 6.9%), myocardial infarction (n = 11; 2.9%), trauma (n = 7; 1.9%), pregnancy (n = 4; 1.1%), asthmatic crisis (n = 4; 1.1%), arrhythmia (n = 3; 0.8%), burns (n = 1; 0.3%), acute episode of cerebrovascular disease (n = 1; 0.3%).

A total of 5022 patients were screened, of which 2510 entered the study. The most common reasons for exclusion were a do-not-resuscitate order (39.5%, n = 980), transfers from other institutions after a length of stay greater than 24 hours (24.9%, n = 626), or some active comorbidity (23.2%, n = 583). The most common site of infection was the urinary tract in 27.8% (n = 692) of cases, followed by pneumonia in 27.5% (n = 690). In 5.4% (n = 135) of the patients, the primary site of infection could not be determined (Figure 1).

The median age of the study patients was 62 years (interquartile range - IQR = 46 - 74), of which 49.8% (n = 1252) were men. The most common comorbidity was kidney disease (22.2%, n = 557), followed by chronic lung disease (19.2%, n = 481). The median Charlson index was 1 (IQR = 0 - 2), the median SOFA score was 4 (IQR = 2 - 6), and the median APACHE score was 14 (IQR = 9 - 18) (Table 1).

**Table 1:** General characteristics of the study population

Variable	Infection site							Total, 2510 (100)
	Urinary tract, 697 (27.8)	Lower respiratory tract, 690 (27.5)	Intra-abdominal, 272 (10.8)	Skin and soft tissues, 238 (9.5)	Bloodstream, 204 (8.1)	Sepsis without focus, 135 (5.4)	Others, 274 (10.9)	
Male sex	301 (43.2)	355 (51.5)	129 (47.4)	142 (59.7)	113 (55.4)	61 (45.2)	150 (54.7)	1251 (49.8)
Age	63 (44 - 76)	65 (53 - 76)	62 (46 - 74)	58 (39 - 67)	58 (45 - 68)	65 (51 - 74)	58 (40 - 70)	62 (46 - 74)
Comorbidities								
Congestive heart failure	46 (6.6)	79 (11.5)	14 (5.2)	24 (10.1)	37 (18.1)	8 (6.0)	14 (5.1)	222 (8.8)
Renal disease	131 (18.8)	119 (17.3)	52 (19.1)	44 (18.5)	131 (64.2)	32 (23.7)	48 (17.5)	557 (22.2)
Any tumor, including leukemia and lymphoma	83 (11.9)	51 (7.4)	42 (15.4)	21 (8.8)	24 (11.8)	18 (13.3)	28 (10.2)	267 (10.6)
Chronic lung disease	96 (13.8)	261 (37.8)	29 (10.7)	22 (9.2)	24 (11.8)	24 (17.8)	25 (9.2)	481 (19.2)
Diabetes with chronic complications	93 (13.4)	76 (11.0)	27 (9.9)	36 (15.1)	52 (25.5)	21 (15.6)	33 (12.0)	338 (13.5)
Diabetes without complications	84 (12.1)	71 (10.3)	34 (12.5)	31 (13.0)	27 (13.2)	9 (6.7)	22 (8.0)	278 (11.1)
AIDS/HIV	4 (0.6)	12 (1.7)	5 (1.8)	5 (2.1)	1 (0.5)	1 (0.7)	10 (3.7)	38 (1.5)
Rheumatologic disease	37(5.3)	37 (5.4)	10 (3.7)	7 (2.9)	10 (4.9)	7 (5.2)	19 (6.9)	127 (5.1)
Metastatic solid tumor	23 (3.3)	11 (1.6)	15 (5.5)	3 (1.3)	4 (2.0)	6 (4.4)	6 (2.2)	68 (2.7)
Drug addiction/Alcoholism	21 (3.0)	42 (6.1)	12 (4.4)	12 (5.0)	4 (2.0)	-	18 (6.6)	109 (4.3)
Organ transplant	42 (6.0)	17 (2.5)	11 (4.0)	5 (2.1)	18 (8.8)	4 (3.0)	14 (5.1)	111 (4.4)
Severity								
Charlson Index	1 (0 - 2)	1 (0 - 2)	1 (0 - 2)	1 (0 - 2)	2 (1 - 3)	1 (0 - 2)	1 (0 - 2)	1 (0 - 2)
Total SOFA Score	3 (2 - 5)	4 (3 - 6)	5 (3 - 6)	2 (1 - 4)	5 (3 - 7)	5 (3 - 7)	4 (2 - 5)	4 (2 - 6)
Total APACHE II	13 (8 - 17)	15 (11 - 19)	13 (9 -17)	10 (6 - 15)	17 (13 - 20)	16 (12 - 20)	13 (8 - 17)	14 (9 - 18)
Septic shock	218 (31.3)	228 (33.0)	122 (44.9)	59 (24.8)	80 (39.2)	66 (48.9)	111 (40.5)	884 (35.2)

AIDS - acquired immunodeficiency syndrome; HIV - human immunodeficiency virus; SOFA - Sequential Organ Failure Assessment; APACHE II - Acute Physiology and Chronic Health Evaluation II. The measurements for continuous variables are the median (IQR) and for categorical: n (%).

### Interventions

A central venous catheter was used in 7.5% of the patients, more commonly in those without focus (11.9%) and less commonly in those with urinary tract infection (3.7%). The median initial central venous pressure was 11 mmHg (IQR = 7 - 14), which was lower in those with sepsis from other sites of infection (8 mmHg, IQR = 6 - 9). The median serum lactate on admission was 2.5mmol/L (IQR = 1.5 - 3.5) and was higher in the group with other sites of infection (2.9mmol/L, IQR = 2.2 - 4, 1). Vasopressors were given to 15.9% of patients; most frequently to those with sepsis without focus (34.1%, n = 46), and least frequently to those with soft tissue infection (7.6%, n = 17). A total of 15.4% of patients required mechanical ventilation, more often those with pneumonia (29.7%, n = 205), followed by patients with sepsis without focus (17.8%, n = 24). Microbiological isolation was obtained for 77.6% of the patients (Table 2).

**Table 2:** Prognostic and treatment variables according to the infection site

Variable	Total, 2510 (100)	Infection site						
		Urinary tract, 697 (27.8)	Lower respiratory tract, 690 (27.5)	Intra-abdominal, 272 (10.8)	Skin and soft tissues, 238 (9.5)	Bloodstream, 204 (8.1)	Sepsis without focus, 135 (5.4)	Others, 274 (10.9)
Lactate on admission*	2381 (94.9)	642 (92.1)	678 (98.3)	246 (90.4)	224 (94.1)	198 (97.1)	133 (98.5)	260 (94.9)
Lactate value on admission*	2.5 (1.5 - 3.5)	2.4 (1.5 - 3.3)	2.3 (1.3 - 3.3)	2.5 (1.5 - 3.9)	2.7 (2.1 - 3.3)	2.6 (1.6 - 3.5)	2.4 (1.4 - 3.7)	2.9 (2.2 - 4.1)
Central venous catheter*	187 (7.5)	26 (3.7)	80 (10.3)	28 (10.3)	12 (5.0)	11 (5.4)	16 (11.9)	14 (5.1)
Initial CVP value**	11 (7 - 14)	8.5 (5 - 15)	12 (7 - 15)	8.5 (4 - 14)	15 (11 - 19)	10 (6 - 17)	8 (0 - 14)	8 (6 - 10)
CVP after 6 hours***	12 (8 - 15)	10 (7 - 12)	13 (8 - 15)	12 (10 - 15)	18 (15 - 20)	13 (10 - 17)	12 (10 - 16)	12 (7 - 14)
IVF in the first 6 hours*	1955 (77.9)	569 (81.6)	484 (70.1)	237 (87.1)	168 (70.6)	153 (75)	117 (86.7)	227 (82.9)
Amount of IVF in the first hour*	1000 (500 - 1500) n = 1184	1000 (500 - 1500) n = 343	1000 (500 - 1100) n = 276	1000 (500 - 1000) n = 177	1000 (300 - 1500) n = 83	1000 (500 - 1500) n = 95	1000 (1000 - 2000) n = 61	1000 (750 - 2000) n = 149
Amount during the first six hours*	1300 (560 - 2000)	1360 (680 - 2000)	1000 (500 - 1600)	1480 (700 - 2035)	1025 (500 - 2000)	1080 (500 - 2000)	1480 (900 - 2360)	1500 (900 - 2500)
IVF ≥ 1500 in the first hour (n = 2510)*	333 (13.3)	96 (13.8)	54 (7.8)	43 (15.8)	28 (13.7)	28 (13.7)	20 (14.8)	62 (22.6)
Vasopressors*	399 (15.9)	83 (11.9)	128 (18.6)	47 (17.3)	18 (7.6)	40 (19.1)	46 (34.1)	37 (13.5)
Blood culture*	2185 (87.1)	612 (87.8)	583 (84.5)	232 (85.3)	185 (77.7)	203 (99.5)	126 (93.3)	244 (89.1)
Cultures taken before starting antibiotics (n = 2185)*	1677 (76.8)	477 (77.9)	424 (72.7)	149 (64.2)	156 (84.3)	167 (82.3)	101 (80.2)	203 (83.2)
Positive result*	635 (29.1)	210 (34.3)	67 (11.5)	77 (33.2)	44 (23.8)	176 (86.7)	3 (2.4)	58 (23.8)
Blood cultures taken in the first 3 hours*	1048 (47.9)	276 (45.1)	266 (45.6)	104 (44.8)	99 (53.5)	109 (53.6)	65 (51.6)	129 (52.9)
Antibiotics in the first 24 hours*	2261 (90.1)	624 (89.5)	649 (94.1)	253 (93)	190 (79.8)	181 (88.7)	125 (92.6)	239 (87.2)
Hours between admission and starting the first AB*	5 (2 - 10)	6 (3 - 10)	5 (2 - 9)	4 (2 - 8)	6 (3 - 12)	5 (2 - 10)	5 (2 - 9)	6 (3 - 10)
Antibiotics administered in the first 3 hours*	790 (34.9)	194 (31.1)	241 (37.1)	108 (42.7)	63 (33.2)	74 (40.9)	40 (32.0)	70 (29.3)
Mechanical ventilation*	387 (15.4)	35 (5.0)	205 (29.7)	42 (15.4)	23 (9.7)	29 (14.2)	24 (17.8)	29 (10.6)

CVP - central venous pressure; IVF - intravenous fluids. The measurements for continuous variables are the median (IQR) and for categorical: n (%).

* p <0.001;

** p >0.05;

*** p <0.05 (continuous variables with the Kruskal-Wallis test and categorical variables with the chi-squared test).

### Outcomes

Overall mortality was 11.5% (n = 289), with the lowest rate in patients with urinary tract infection (5.0%, n = 35). The sites of infection in which there was greater mortality were pneumonia (17.5%, n = 121), sepsis without focus (15.6%, n = 21), and bloodstream infection (14.7%, n = 30). A total of 42.3% of the patients were transferred to ICU, more often those with sepsis without focus (63.7%, n = 86). The median length of hospital stay for patients who were discharged was 10 days (IQR 6 - 17); this was higher for patients with skin and soft tissue foci (13 days, IQR 8 - 22) and bloodstream focus (13 days, IQR 9 - 20). For patients who died, the median length-of-stay was 9 days (IQR 3 - 16), and this was longer in patients with intra-abdominal infection (14 days, IQR 5 - 23) and shorter in patients with sepsis without focus (2 days, IQR 1 - 7) (Table 3).

**Table 3:** Outcomes according to the site of infection

Variable	Total, 2510 (100)	Infection site							p value
		Urinary tract, 697 (27.8)	Lower respiratory tract, 690 (27.5)	Intra-abdominal, 272 (10.8)	Skin and soft tissues, 238 (9.5)	Bloodstream, 204 (8.1)	Sepsis without focus, 135 (5.4)	Others, 274 (10.9)	
Mortality	289 (11.5)	35 (5.0)	121 (17.5)	38 (14.0)	19 (8.0)	30 (14.7)	21 (15.6)	25 (9.1)	< 0.001
Transfer to ICU	1062 (42.3)	207 (29.7)	346 (50.1)	150 (55.2)	67 (28.2)	92 (45.1)	86 (63.7)	114 (41.6)	< 0.001
Hospital stay in patients who were discharged	10 (6 - 17)	9 (5 - 14)	10 (6 - 17)	11 (7 - 18)	13 (8 - 22)	13 (9 - 20)	11 (8 - 18)	8 (5 - 15)	< 0.001
Hospital stay in patients who died	9 (3 - 16)	11 (6 - 16)	10 (3 - 17)	14 (5 - 23)	11 (5 - 21)	5 (1 - 15)	2 (1 - 7)	4 (2 - 16)	0.0029

ICU - intensive care unit. The measurements for continuous variables are the median (IQR) and for categorical: n (%).

The group of sepsis due to urinary tract infection was taken as the reference group. In univariate analysis, the other sites of infection were associated with a significant increase in mortality, except for the skin and soft tissue group (OR 1.5; 95%CI, 0.9 - 2.9). The highest risk occurred in the pneumonia group (OR 4; 95%CI, 2.7 - 6), followed by sepsis without focus (OR 3.5; 95%CI, 2 - 6.2) and bloodstream infection (OR 3.3; 95%CI, 1.9 - 5.5). These associations were maintained when performing multivariate analysis adjusting for confounding variables, including lactate, SOFA, and APACHE II scores, with the pneumonia group having the highest risk (OR 3.4; 95%CI, 2.2 - 5.2), followed by the skin and soft tissue focus group (OR 2.6; 95%CI, 1.4 - 5.0) (Table 4). Regarding the risk of admission to the ICU, in univariate analysis, the group of patients with the highest risk was that of sepsis without focus (OR 4.2; 95%CI, 2.8 - 6.1), followed by the intra-abdominal infection group (OR 3; 95%CI, 2.1 - 3.9) and pneumonia group (OR 2.4; 95%CI, 1.9 - 3), without finding a significant association between this outcome and the skin and soft tissue infection source (OR 0.9; 95%CI, 0.7 - 1.3). When SOFA, APACHE II score and lactate were included in the multivariate model, the association between bloodstream infection and admission to the ICU, such as that of the "other infections" group and admission to the ICU, lost statistical significance (OR 0.9; 95%CI, 0.7 - 1.4 and OR 1.3; 95%CI, 0.9 - 1.8, respectively), while the other groups maintained statistical significant association (Table 5).

**Table 4:** Sequential univariate and multivariate logistic regression for mortality

Infection site	Univariate		Multivariate*		Multivariate¥		Multivariate£	
	OR (95%CI)	p value	OR (95%CI)	p value	OR (95%CI)	p value	OR (95%CI)	p value
Urinary tract	1.0 (reference)		1.0 (reference)		1.0 (reference)		1.0 (reference)	
Pneumonia	4 (2.7 - 6)	< 0.001	3.8 (2.6 - 5.8)	< 0.001	3.9 (2.6 - 5.8)	< 0.001	3.4 (2.2 - 5.2)	< 0.001
Intra-abdominal	3.1 (1.9 - 5)	< 0.001	3.2 (1.9 - 5.1)	< 0.001	3.1 (1.9 - 5.0)	< 0.001	1.9 (1.1 - 3.3)	0.024
Skin and soft tissues	1.6 (0.9 - 2.9)	0.094	1.8 (0.9 - 3.2)	0.050	1.8 (1.0 - 3.2)	0.048	2.6 (1.4 - 5.0)	0.003
Bloodstream	3.3 (1.9 -5.5)	< 0.001	3.4 (2 - 5.7)	< 0.001	3.2 (1.9 - 5.3)	< 0.001	2.0 (1.1 - 3.6)	0.018
Sepsis without focus	3.5 (2.0 - 6.2)	< 0.001	3.4 (1.9 - 6.1)	< 0.001	3.3 (1.9 - 5.9)	< 0.001	2.0 (1.1 - 3.8)	0.028
Other infections	1.9 (1.1 - 3.2)	0.018	2 (1.2 - 3.5)	0.009	2 (1.1 - 3.4)	0.014	1.5 (0.8 - 2.8)	0.175

* Model including the variables: age, sex, and Charlson index.

¥ Model including the variables: age, sex, Charlson index, IVF ≥ 1500 first hour, antibiotics in the first 3 hours, blood cultures in the first 3 hours.

£ Model including the variables: age, sex, Charlson index, IVF ≥ 1500 first hour, antibiotics in the first 3 hours, blood cultures in the first 3 hours, lactate, SOFA score, and APACHE II score.

**Table 5:** Sequential univariate and multivariate logistic regression for admission to the ICU

Infection site	Univariate		Multivariate*		Multivariate¥		Multivariate£	
	OR (95%CI)	p value	OR (95%CI)	p value	OR (95%CI)	p value	OR (95%CI)	p value
Urinary tract	1.0 (reference)		1.0 (reference)		1.0 (reference)		1.0 (reference)	
Pneumonia	2.4 (1.9 - 3)	< 0.001	2.4 (1.9 - 3.0)	< 0.001	2.5 (1.9 - 3.1)	< 0.001	1.8 (1.4 - 2.4)	< 0.001
Intra-abdominal	3 (2.1 - 3.9)	< 0.001	2.9 (2.2 - 3.9)	< 0.001	2.9 (2.1 - 3.9)	< 0.001	2.1 (1.5 - 3.0)	< 0.001
Skin and soft tissues	0.9 (0.7 - 1.3)	0.651	0.9 (0.7 - 1.3)	0.642	0.9 (0.7 - 1.3)	0.683	1.2 (0.8 - 1.8)	0.268
Bloodstream	1.9 (1.4 - 2.7)	< 0.001	2.0 (1.4 - 2.7)	< 0.001	1.8 (1.3 - 2.6)	< 0.001	0.9 (0.6 - 1.4)	0.706
Sepsis without focus	4.2 (2.8 - 6.1)	< 0.001	4.2 (2.8 - 6.2)	< 0.001	4.2 (2.8 - 6.2)	< 0.001	2.4 (1.6 - 3.8)	< 0.001
Other infections	1.7 (1.3 - 2.3)	< 0.001	1.7 (1.3 - 2.2)	< 0.001	1.6 (1.2 - 2.1)	0.002	1.3 (0.9 - 1.8)	0.147

* Model including the variables: age, sex, and Charlson index.

¥ Model including the variables: age, sex, Charlson index, IVF ≥ 1500 first hour, antibiotics in the first 3 hours, blood cultures in the first 3 hours.

£ Model including variables: age, sex, Charlson index, IVF ≥ 1500 first hour, antibiotics in the first 3 hours, blood cultures in the first 3 hours, lactate, SOFA score, and APACHE II score.

## DISCUSSION

Sepsis is a heterogeneous syndrome caused by various microorganisms, comprising clinical parameters determined by infections in different anatomical sites and occurring in hosts with variable immune responses to a similar aggression. In this multicenter cohort of patients admitted to the emergency department with suspected septic shock or severe sepsis, we found significant differences in the risk of in-hospital mortality and admission to the ICU according to the site of infection, with the lowest risk in the urinary tract infection group, which mostly persisted even after adjusting for comorbidities, early interventions, and various severity markers.

The lower mortality observed in the group of patients with urinary tract infection in this study is consistent with previous studies.^(^^19-22^^)^ A systematic review that analyzed 19 studies evaluating the association between site of infection and mortality also found that patients with pneumonia had a consistently higher risk of mortality, and those with urinary tract infection had a consistently lower risk. However, this same review also showed that the results were not conclusive enough to determine the impact of the site of infection on the risk of death, and a meta-analysis was not performed due to heterogeneity between the studies.^(^^9^^)^ On the other hand, and in disagreement with our findings, a prospective observational study including 3588 patients with sepsis and septic shock found no association between site of infection or the isolated microorganism and in-hospital mortality.^(^^23^^)^

Although pneumonia had the greatest association as a prognostic factor in our study, it is important to note the relevance of the group of sepsis without focus. This group of patients represents a clinical challenge from a diagnostic and therapeutic viewpoint, which is reflected in the increased risk of death and admission to the ICU that was found even after adjusting for multiple confounding variables. To the best of our knowledge, similar results have only been described in a retrospective study with 248 patients, which found that those patients without specific focus on admission or with multiple sites of infection had higher mortality during hospitalization.^(^^23^^)^ High mortality in this group could be explained by two reasons: an inadequate empirical antibiotic therapy or an incorrect diagnosis of sepsis. The selection of appropriate antibiotic treatment must consider aspects such as the local prevalence and resistance profile, the patient's comorbidities, and the anatomical site of infection.^(^^24,25^^)^ In patients without specific focus, in addition to the difficulties inherent in the uncertainty of the source of infection to determine antibiotic therapy, the start of treatment can be delayed, increasing the risk of death.^(^^25-27^^)^ In our analysis, this circumstance was considered when adjusting for the early introduction of antibiotics in multivariate analysis, and the association did not lose statistical significance.

On the other hand, since the diagnosis of sepsis is basically clinical, patients with noninfectious etiologies simulating this condition could be incorrectly classified as septic. A study found that 18% of patients admitted to the emergency department with a diagnosis of severe sepsis had a final diagnosis of noninfectious disease, such as inflammatory bowel disease, acute heart failure, systemic lupus erythematosus, or adverse drug effects, reaching 48% in patients from whom no positive cultures were obtained.^(^^28^^)^ The percentage of positive blood cultures in our study was similar to that reported in previous studies.^(^^29,30^^)^ Similarly, it has been described that patients with sepsis without focus and negative cultures have lower mortality than patients with positive cultures;^(^^31^^)^ however, our findings, which we have corroborated in another recently published study, did not agree with this statement.^(^^32^^)^

Different scores and models have been used to determine the prognosis of patients with sepsis; however, none of them consider the individual characteristics of the patient in terms of their infection, making it possible to overlook influential variables. Recently, quick SOFA (qSOFA) was proposed as a model for the identification of patients at risk of poor prognosis,^(^^2^^)^ with extremely variable results in its validation^(^^33^^)^ even in studies conducted specifically in the emergency department^(^^34-36^^)^ and in our region.^(^^37^^)^ Although the criteria of the systemic inflammatory response syndrome (SIRS) have been associated with poor prognostic performance in patients with sepsis,^(^^38^^)^ in recent meta-analyses, qSOFA was inferior to SIRS in terms of the diagnosis of sepsis and prediction of mortality.^(^^39,40^^)^ It could be assumed that the importance of site of infection is due to the specific needs for interventions and the progression of organ dysfunction, which differ widely according to the focus. However, we performed a sequential multivariable logistic regression, initially including demographic variables, then adding interventions, and finally severity markers, and in most cases the significant association between focus and mortality was maintained. Our results acquire relevance as far as they help to elucidate the relationship between site of infection and adverse outcomes in patients with sepsis, a clinical situation suggesting that prognostic models should be developed and validated in a stratified manner independently for each site of infection.

Regarding interventions, 78% of the participants received intravenous fluids in the first 6 hours, and only 34.9% were given antibiotics in the first 3 hours, which represents low adherence to the international proposals for the treatment of sepsis and septic shock. However, the overall mortality of the participants was lower than that reported in other studies in patients with a similar diagnosis.^(^^22,38,41,42^^)^ It might be possible that for unknown reasons our population had an intrinsically lower risk of death, but it must be borne in mind that a large part of the studies reporting higher mortality are randomized clinical trials or observational studies conducted in the ICU, while ours was conducted in the emergency department, which could explain the differences that were found.

The strengths of this study are the sample size, the variables used, the prospective design, and the multivariate analysis, which together make this study, according to our knowledge, the first of its kind to describe these associations. The main limitation is that it was a secondary analysis and therefore was not specifically designed to identify the association of interest. Since those patients who were taken to surgery in the first 24 hours were excluded from the original cohort, their data were not included in our study, raising the possibility of excluding patients with sepsis who required urgent surgical intervention to control the infection, which could affect our results. Additionally, it is important to note that patients were included according to the definitions of the second international consensus (Sepsis-2) for what was then called severe sepsis and septic shock; therefore, some characteristics of this study population, according to the latest consensus (Sepsis-3), may affect its applicability and generalization at present.

## CONCLUSION

There is a significant and independent association between the site of infection and in-hospital mortality in patients with sepsis or septic shock. Urinary tract infection had the lowest risk of death or admission to the intensive care unit. The above should be considered in the development of prognostic models, aiming to improve the care and treatment of these patients.
